# Testing the accuracy of low-beam-energy electron-excited X-ray microanalysis with energy-dispersive spectrometry

**DOI:** 10.1007/s10853-024-10285-4

**Published:** 2024-10-14

**Authors:** Dale E. Newbury, Nicholas W. M. Ritchie

**Affiliations:** National Institute of Standards and Technology, Gaithersburg, MD 20899-8370 USA

## Abstract

The accuracy of electron-excited X-ray microanalysis with energy-dispersive spectrometry (EDS) has been tested in the low beam energy range, specifically at an incident beam energy of 5 keV, which is the lowest beam energy for which a useful characteristic X-ray peak can be excited for all elements of the periodic table, excepting H and He. Elemental analysis results are reported for certified reference materials (CRM), stoichiometric compounds, minerals, and metal alloys of independently known or measured composition which had microscopic homogeneity suitable for microanalysis. Two-hundred sixty-three concentration measurements for 39 elements in 113 materials were determined following the *k-ratio protocol* and using the EDS analytical software NIST DTSA-II. The accuracy of the results, as characterized by the *relative deviation from expected value* (RDEV) metric, was such that more than 98% of the results were found to be captured within a range of ±5% RDEV, while 82% of the results fell in the range -2% to 2% RDEV.

## Introduction

Electron-excited X-ray microanalysis with energy-dispersive spectrometry (EDS) is a spatially resolved elemental characterization technique that is widely applied in the physical, biological, and forensic sciences, technology, failure analysis, etc. [1]. “Conventional” analytical strategy involves selecting the incident beam energy, E_0_, to be 10 keV or higher to excite analytically useful characteristic X-rays from all elements of the periodic table, with the exception of H and He, which do not produce characteristic X-rays. Because of the strong dependence of the electron range on the incident beam energy, $R \approx {{E}_{0}}^{1.66}$, the spatial resolution of the measurement can be significantly improved by reducing the beam energy to the lowest value compatible with achieving useful X-ray excitation for all elements to be measured in the specimen. As detailed in a previous paper [2], “low-beam-energy microanalysis” involves choosing the beam energy in the range E_0_ ≤ 5 keV. E_0_ = 5 keV is the lowest beam energy for which an analytically useful characteristic X-ray can be excited for all elements except H and He. However, even with a choice of E_0_ = 5 keV, less familiar low photon energy characteristic X-rays must be used for several elements, e.g., the Ti L-family (≈ 0.45 keV) instead of the Ti K-family (4.5 keV) and the Ba M-family (0.6–1.1 keV) instead of the Ba L-family (4.467 keV). As the beam energy is lowered below 5 keV, elements are progressively lost to analysis due to inadequate or no excitation [2].

When an unknown material is analyzed, the absolute accuracy of the analytical results cannot be known due to uncertainty in the various parameters necessary to calculate the matrix correction factors. Robust analytical practice requires that an uncertainty budget be associated with each reported elemental concentration, such as that provided by the NIST DTSA-II software platform [3]. DTSA-II attaches to each concentration value an uncertainty budget that includes the contribution of the random component due to the characteristic X-ray measurement statistics (from the unknown and the standard) as well as an estimation of the systematic components that arise from the principal matrix correction corrections for electron scattering and energy loss (the “Z” factor) and the self-absorption of X-rays (the “A” factor). This uncertainty budget can then be used to describe a “confidence range” within which the true concentration value can be expected to reside [4–6].

The general ”accuracy” of electron-excited X-ray microanalysis can be estimated by analyzing “challenge specimens” whose compositions are known from independent analysis so that the measured value can be reasonably compared to a known reference value. A limited number of microanalysis-qualified certified reference materials (CRM) are available from national measurement institutions, such as the National Institute of Standards and Technology (U.S.A.). Additional challenge specimens can be derived from materials whose composition is sharply constrained by their stoichiometric nature and which do not exhibit a range of solid solubility, e.g., compounds such as FeS_2_, CuS, Fe_2_O_3_, etc. A second critical criterion is that these challenge specimens must also be homogeneous on a microscopic scale, a condition which can be confirmed by a systematic survey of the material by “point beam” random sampling or by area scanning /compositional mapping to examine microstructural compositional details. The current study has undertaken analysis of a wide range of challenge specimens to develop an analytical history sufficient to make an estimate of the accuracy of electron-excited X-ray microanalysis with energy-dispersive spectrometry for a beam energy of E_0_ = 5 keV. Analysis followed the standards-based measurement protocol, in which each element in the sample is measured relative to that same element present at a known concentration in a “standard”, which can be a pure element, a binary stoichiometric compound, or an elemental mixture such as a glass available as a CRM [1].

## Materials and methods

All materials analyzed are listed individually in Table 1, summary of results. Materials and sources analyzed in this study include:

1. National Institute of Standards and Technology (NIST) Standard Reference Materials (SRM) specially developed to be homogeneous on the microscopic scale so as to be suitable for microanalysis applications:
  1. SRM 470 K411 glass (O-Mg-Si-Ca-Fe)
  (2) SRM 470 K412 glass (O-Mg-Al-Si-Ca-Fe)
  (3) SRM 479 (Fe-Cr-Ni Stainless Steel)
  (4) SRM 481, Gold-Silver alloys (nominal 20, 40, 60, and 80 weight percent)
  (5) SRM 482, Gold-Copper alloys (nominal 20, 40, 60, and 80 weight percent)
  (6) SRM 1871 K456 glass (O-Si-Pb)
  (7) SRM 1872 K453 glass (O-Ge-Pb)
  (8) SRM 1873 K458 glass (O-Si-Zn-Ba)
  (9) SRM 1875 K496 glass (O-Mg-Al-P)
2. European Commission, Community Bureau of Reference
  1. Fe_3_C (CRM BCR-726)

3. Reagent compounds, e.g., BaTiO_3_, PbS, MoS_2_ (source: Alfa Aesar, Tewksbury, MA 01876)).
4. Stoichiometric binary compounds, e.g., TiN, TiO_2_, Cr_2_N, etc., with surfaces prepared for microanalysis (source: Geller Microanalytical Laboratory, Topfield, MA 01983)
5. Minerals of known composition and micro-homogeneity, e.g., albite, arsenopyrite, calcite, chalcopyrite, cinnabar, cryolite, dolomite, fluorapatite, galena, jadeite, kyanite, hematite, magnetite, pyrite, rhodonite, scheelite, willemite, wollastonite, zircon (source: SPI (West Chester, PA, 19381-0656, USA) )

**Table 1:** Compilation of Analytical Results for Analysis at E_0_ = 5 keV

Row	Material	Material number	Element	Known mass conc	Analytical platform	0.1-5 keV counts	Standard	Raw total	Raw mass conc	DTSA_II Unc_Budget	Raw -conc RDEV %	Normalized mass conc	Norm-conc RDEV %	Atomic conc reference	Atomic conc	Atom-conc RDEV %
1	SRM482_Au20Cu80_5kV	1	Cu	0.7985	JEOL8500f-BrukerQuad	6,705,457	Cu	1.0066	0.8120	±0.0117	1.7	0.8067	1.0	0.9248	0.9282	0.37
2	SRM482_Au20Cu80_5kV	1	Au	0.2012	JEOL8500f-BrukerQuad	6,705,457	Au	1.0066	0.1946	±0.0026	-3.3	0.1933	-3.9	0.0752	0.0718	-4.5
3	SRM482_Au40Cu60_5kV	2	Cu	0.5992	JEOL8500f-BrukerQuad	6,532,544	Cu	1.0078	0.6160	±0.0175	2.8	0.6112	2.0	0.8224	0.8297	0.89
4	SRM482_Au40Cu60_5kV	2	Au	0.4010	JEOL8500f-BrukerQuad	6,532,544	Au	1.0078	0.3918	±0.0040	-2.3	0.3888	-3.0	0.1776	0.1703	-4.1
5	SRM482_Au60Cu40_5kV	3	Cu	0.3964	JEOL8500f-BrukerQuad	6,376,277	Cu	0.9975	0.4128	±0.0177	4.2	0.4138	4.4	0.6706	0.6863	2.3
6	SRM482_Au60Cu40_5kV	3	Au	0.6036	JEOL8500f-BrukerQuad	6,376,277	Au	0.9975	0.5848	±0.0043	-3.1	0.5862	-2.9	0.3294	0.3137	-4.8
7	SRM482_Au80Cu20_5kV	4	Cu	0.1983	JEOL8500f-BrukerQuad	6,305,887	Cu	0.9868	0.2086	±0.0122	5.2	0.2114	6.6	0.4340	0.4538	4.6
8	SRM482_Au80Cu20_5kV	4	Au	0.8015	JEOL8500f-BrukerQuad	6,305,887	Au	0.9868	0.7782	±0.0031	-2.9	0.7886	-1.6	0.5660	0.5462	-3.5
9	SRM481_Au20Ag80_5kV	5	Ag	0.7758	JEOL8500f-BrukerQuad	4,778,550	Ag	1.013	0.7841	±0.0054	1.1	0.7738	-0.26	0.8633	0.8620	-0.16
10	SRM481_Au20Ag80_5kV	5	Au	0.2243	JEOL8500f-BrukerQuad	4,778,550	Au	1.013	0.2292	±0.0027	2.2	0.2262	0.86	0.1367	0.1380	0.98
11	SRM481_Au40Ag60_5kV	6	Ag	0.5993	JEOL8500f-BrukerQuad	5,070,877	Ag	1.002	0.6084	±0.0068	1.5	0.6073	1.3	0.7322	0.7385	0.86
12	SRM481_Au40Ag60_5kV	6	Au	0.4003	JEOL8500f-BrukerQuad	5,070,877	Au	1.002	0.3934	±0.0036	-1.7	0.3927	-1.9	0.2678	0.2615	-2.3
13	SRM481_Au60Ag40_5kV	7	Ag	0.3992	JEOL8500f-BrukerQuad	5,486,996	Ag	0.9991	0.4057	±0.0069	1.6	0.4061	1.7	0.5483	0.5552	1.3
14	SRM481_Au60Ag40_5kV	7	Au	0.6005	JEOL8500f-BrukerQuad	5,486,996	Au	0.9991	0.5934	±0.0038	-1.2	0.5939	-1.1	0.4517	0.4448	-1.5
15	SRM481_Au80Ag20_5kV	8	Ag	0.1996	JEOL8500f-BrukerQuad	6,015,553	Ag	1.005	0.2040	±0.0047	2.2	0.2030	1.7	0.3129	0.3175	1.5
16	SRM481_Au80Ag20_5kV	8	Au	0.8005	JEOL8500f-BrukerQuad	6,015,553	Au	1.005	0.8006	±0.0029	0.01	0.7970	-0.44	0.6871	0.6825	-0.67
17	SRM479_Stainless_Steel_5keV	9	Cr	0.183	JEOL8500f-BrukerQuad	6,491,256	Cr	1.085	0.2038	±0.0181	11.4	0.1879	2.7	0.1949	0.2001	2.7
18	SRM479_Stainless_Steel_5keV	9	Fe	0.710	JEOL8500f-BrukerQuad	6,491,256	Fe	1.085	0.7677	±0.0424	8.1	0.7078	-0.31	0.7041	0.7016	-0.36
19	SRM479_Stainless_Steel_5keV	9	Ni	0.107	JEOL8500f-BrukerQuad	6,491,256	NiSi	1.085	0.1131	±0.0108	5.7	0.1043	-2.5	0.1010	0.0984	-2.6
20	CuO_5kV	10	O	0.2011	JEOL8500f-BrukerQuad	9,844,586	MgO	0.9793	0.1915	±0.0012	-4.8	0.1956	-2.7	0.5000	0.4913	-1.7
21	CuO_5kV	10	Cu	0.7989	JEOL8500f-BrukerQuad	9,844,586	Cu	0.9793	0.7878	±0.0032	-1.4	0.8044	0.69	0.5000	0.5087	1.7
22	Cr2O3_5keV	11	O	0.3158	JEOL8500f-BrukerQuad	5,268,245	O	0.9008	0.2888	±0.0098	-8.6	0.3205	1.5	0.6000	0.6053	0.88
23	Cr2O3_5keV	11	Cr	0.6642	JEOL8500f-BrukerQuad	5,268,245	Cr	0.9008	0.6121	±0.0306	-7.8	0.6795	2.3	0.4000	0.3947	-1.3
24	Fe3C_5kV	12	C	0.0669	JEOL8500f-BrukerQuad	3,585,708	C	1.059	0.0688	±0.0155	2.8	0.0649	-7.2	0.2500	0.2438	-2.5
25	Fe3C_5kV	12	Fe	0.9331	JEOL8500f-BrukerQuad	3,585,708	Fe	1.059	0.9916	±0.0044	6.3	0.9351	0.21	0.7500	0.7562	0.83
26	NiTi_5kVA	13	Ti	0.4492	JEOL8500f-BrukerQuad	12,088,360	Ti	1.192	0.5192	±0.0394	15.6	0.4356	-3.0	0.5000	0.4862	-2.8
27	NiTi_5kV	13	Ni	0.5508	JEOL8500f-BrukerQuad	12,088,360	Ni	1.192	0.6729	±0.0232	22.2	0.5644	2.5	0.5000	0.5138	2.8
28	NiSi_5kV	14	Si	0.3236	JEOL8500f-BrukerQuad	7,329,688	Si	0.9857	0.3182	±0.0026	-1.6	0.3226	-0.31	0.5000	0.4988	-0.23
29	NiSi_5kV	14	Ni	0.6764	JEOL8500f-BrukerQuad	7,329,688	NiTi	0.9857	0.6681	±0.0151	-1.2	0.6774	0.15	0.5000	0.5012	0.23
30	NiSi2_5kV	15	Si	0.4890	JEOL8500f-BrukerQuad	5,908,406	Si	1.065	0.5094	±0.0030	4.2	0.4785	-2.1	0.6667	0.6573	-1.4
31	NiSi2_5kV	15	Ni	0.5110	JEOL8500f-BrukerQuad	5,908,406	NiTi	1.065	0.5551	±0.0097	8.6	0.5215	2.1	0.3333	0.3427	2.8
32	TiB2_5kV	16	B	0.3112	JEOL8500f-BrukerQuad	4,130,925	B	1.174	0.3517	±0.0495	14.8	0.3040	-2.3	0.6667	0.6591	-1.1
33	TiB2_5kV	16	Ti	0.6888	JEOL8500f-BrukerQuad	4,130,925	Ti	1.174	0.8171	±0.0251	18.6	0.6960	1.0	0.3333	0.3409	2.3
34	TiSi2_5kV	17	Si	0.5399	JEOL8500f-BrukerQuad	6,011,950	Si	0.9919	0.5437	±0.0004	0.70	0.5481	1.5	0.6667	0.6739	1.1
35	TiSi2_5kV	17	Ti	0.4601	JEOL8500f-BrukerQuad	6,011,950	Ti	0.9919	0.4438	±0.0256	-2.6	0.4519	-1.8	0.3333	0.3261	-2.2
36	TiN_5kV	18	N	0.2264	JEOL8500f-BrukerQuad	2,518,085	GaN	1.207	0.2695	±0.0832	19.0	0.2233	-1.4	0.5000	0.4955	-0.90
37	TiN_5kV	18	Ti	0.7736	JEOL8500f-BrukerQuad	2,518,085	TiSi2	1.207	0.9375	±0.0763	21.1	0.7767	0.40	0.5000	0.5045	0.90
38	TiO2_5kV	19	O	0.4007	JEOL8500f-BrukerQuad	5,391,466	O	0.9903	0.3961	±0.0305	-1.1	0.4000	-0.17	0.6667	0.6661	-0.10
39	TiO2_5kV	19	Ti	0.5993	JEOL8500f-BrukerQuad	5,391,466	Ti	0.9903	0.5942	±0.0115	-0.85	0.6000	0.12	0.3333	0.3339	0.19
40	Al3Ni_5kV	20	Al	0.5797	JEOL8500f-BrukerQuad	7,511,728	Al	1.06	0.6173	±0.0046	6.5	0.5821	0.41	0.7500	0.7518	0.24
41	Al3Ni_5kV	20	Ni	0.4203	JEOL8500f-BrukerQuad	7,511,728	NiTi	1.06	0.4432	±0.0087	5.5	0.4179	-0.57	0.2500	0.2482	-0.73
42	Al3Ni2_5kV	21	Al	0.4081	JEOL8500f-BrukerQuad	7,732,677	Al2O3	1.028	0.4313	±0.0047	5.7	0.4195	2.80	0.6000	0.6112	1.9
43	Al3Ni2_5kV	21	Ni	0.5919	JEOL8500f-BrukerQuad	7,732,677	NiTi	1.028	0.5969	±0.0110	0.91	0.5805	-1.90	0.4000	0.3888	-2.8
44	50Fe-50Cr_5kV	22	Cr	0.4984	JEOL8500f-BrukerQuad	5,937,198	Cr	1.053	0.5243	±0.0275	5.2	0.4978	-0.12	0.5175	0.5156	-0.36
45	50Fe-50Cr_5kV	22	Fe	0.4991	JEOL8500f-BrukerQuad	5,937,198	Fe3C	1.053	0.5289	±0.0571	6.0	0.5022	0.62	0.4825	0.4844	0.39
46	AlN_5keV	23	N	0.3417	JEOL8500f-BrukerQuad	3,807,755	GaN	1.04	0.3626	±0.0573	6.1	0.3487	2.0	0.5000	0.5077	1.5
47	AlN_5keV	23	Al	0.6583	JEOL8500f-BrukerQuad	3,807,755	Al	1.04	0.6773	±0.0013	2.9	0.6513	-1.1	0.5000	0.4923	-1.5
48	BaSi2O5_Sanbornite	24	O	0.2925	JEOL8500f-BrukerQuad	7,412,983	MgO	0.9904	0.2865	±0.0097	-2.1	0.2893	-1.1	0.6250	0.6183	-1.1
49	BaSi2O5_Sanbornite	24	Si	0.2054	JEOL8500f-BrukerQuad	7,412,983	Si	0.9904	0.2092	±0.0015	1.9	0.2113	2.9	0.2500	0.2573	2.9
50	BaSi2O5_Sanbornite	24	Ba	0.5021	JEOL8500f-BrukerQuad	7,412,983	BaCO3	0.9904	0.4946	±0.0068	-1.5	0.4994	-0.53	0.1250	0.1255	-0.50
51	Cr2B	25	B	0.0942	JEOL8500f-BrukerQuad	3,257,829	B	0.9711	0.0882	±0.0372	-6.4	0.0908	-3.6	0.3333	0.3244	-2.7
52	Cr2B	25	Cr	0.9058	JEOL8500f-BrukerQuad	3,257,829	Cr	0.9711	0.8829	±0.0106	-2.5	0.9092	0.38	0.6667	0.6756	1.3
53	CrB	26	B	0.1721	JEOL8500f-BrukerQuad	3,084,682	B	0.9996	0.1630	±0.0649	-5.3	0.1631	-5.2	0.5000	0.4839	-3.2
54	CrB	26	Cr	0.8279	JEOL8500f-BrukerQuad	3,084,682	Cr	0.9996	0.8366	±0.0177	1.1	0.8369	1.1	0.5000	0.5161	3.2
55	CrB2	27	B	0.2937	JEOL8500f-BrukerQuad	2,954,279	B	0.9626	0.2728	±0.0988	-7.1	0.2834	-3.5	0.6667	0.6554	-1.7
56	CrB2	27	Cr	0.7063	JEOL8500f-BrukerQuad	2,954,279	Cr	0.9626	0.6898	±0.0260	-2.3	0.7166	1.5	0.3333	0.3446	3.4
57	YBa2Cu3O7_xtal	28	O	0.1681	JEOL8500f-BrukerQuad	8,181,180	BaCO3	1.019	0.1646	±0.0118	-2.1	0.1615	-3.9	0.5385	0.5264	-2.2
58	YBa2Cu3O7_xtal	28	Cu	0.2862	JEOL8500f-BrukerQuad	8,181,180	CuS	1.019	0.2972	±0.0183	3.8	0.2916	1.9	0.2308	0.2393	3.7
59	YBa2Cu3O7_xtal	28	Y	0.1335	JEOL8500f-BrukerQuad	8,181,180	Y2O3	1.019	0.1315	±0.0008	-1.5	0.1290	-3.4	0.0769	0.0757	-1.6
60	YBa2Cu3O7_xtal	28	Ba	0.4123	JEOL8500f-BrukerQuad	8,181,180	BaCO3	1.019	0.4260	±0.0097	3.3	0.4179	1.4	0.1538	0.1587	3.2
61	MoC	29	C	0.1113	JEOL8500f-BrukerQuad	4,100,229	C	1.061	0.1139	±0.0317	2.4	0.1074	-3.5	0.5000	0.4901	-2.0
62	MoC	29	Mo	0.8887	JEOL8500f-BrukerQuad	4,100,229	Mo	1.061	0.9470	±0.0017	6.6	0.8926	0.44	0.5000	0.5099	2.0
63	As2Te3_5kV	30	As	0.2813	JEOL8500f-Bruker2019	5,212,612	As	1.114	0.3144	±0.0066	11.8	0.2821	0.28	0.4000	0.4009	0.23
64	As2Te3_5kV	30	Te	0.7187	JEOL8500f-Bruker2019	5,212,612	Te	1.114	0.8000	±0.0093	11.3	0.7179	-0.11	0.6000	0.5991	-0.15
65	Bi2Se3_5kV	31	Se	0.3617	JEOL8500f-Bruker2019	6,505,288	Se	1.009	0.3644	±0.0035	0.75	0.3610	-0.19	0.6000	0.5993	-0.12
66	Bi2Se3_5kV	31	Bi	0.6383	JEOL8500f-Bruker2019	6,505,288	Bi	1.009	0.6449	±0.0032	1.0	0.6390	0.11	0.4000	0.4007	0.18
67	CdS_5kV	32	S	0.2219	JEOL8500f-Bruker2019	4,535,223	CuS	1.0386	0.2236	±0.0011	0.77	0.2153	-3.0	0.5000	0.4902	-2.0
68	CdS_5kV	32	Cd	0.7781	JEOL8500f-Bruker2019	4,535,223	Cd	1.0386	0.8150	±0.0036	4.7	0.7847	0.85	0.5000	0.5098	2.0
69	CdSe_5kV	33	Se	0.4126	JEOL8500f-Bruker2019	5,493,373	Se	1.043	0.4167	±0.0045	0.99	0.3995	-3.2	0.5000	0.4864	-2.7
70	CdSe_5kV	33	Cd	0.5874	JEOL8500f-Bruker2019	5,493,373	Cd	1.043	0.5874	±0.0025	6.7	0.6005	3.0	0.5000	0.5136	2.7
71	Cu2O_5kV	34	O	0.1118	JEOL8500f-Bruker2019	7,363,940	MgO	1.0303	0.1131	±0.0052	1.2	0.1097	-1.9	0.3333	0.3287	-1.4
72	Cu2O_5kV	34	Cu	0.8882	JEOL8500f-Bruker2019	7,363,940	CuS	1.0303	0.9173	±0.0136	3.3	0.8903	0.24	0.6667	0.6713	0.69
73	CuS_5keV	35	S	0.3354	JEOL8500f-Bruker2019	6,343,848	ZnS	0.974	0.3258	±0.0020	-2.9	0.3345	-0.3	0.5000	0.4990	-0.20
74	CuS_5keV	35	Cu	0.6646	JEOL8500f-Bruker2019	6,343,848	Cu2O	0.974	0.6482	±0.0101	-2.5	0.6655	0.14	0.5000	0.5010	0.20
75	FeS_5keV	36	S	0.3647	JEOL8500f-Bruker2019	4,590,580	CuS	1.0191	0.3698	±0.0018	1.4	0.3629	-0.49	0.5000	0.4980	-0.40
76	FeS_5keV	36	Fe	0.6353	JEOL8500f-Bruker2019	4,590,580	Fe	1.0191	0.6492	±0.0343	3.6	0.6371	0.28	0.5000	0.5020	0.40
77	FeS2_5keV	37	S	0.5345	JEOL8500f-BrukerQuad	4,624,933	CuS	1.0234	0.5577	±0.0024	4.3	0.5449	1.9	0.6667	0.6759	1.4
78	FeS2_5keV	37	Fe	0.4655	JEOL8500f-BrukerQuad	4,624,933	FeAl3	1.0234	0.4658	±0.0091	0.06	0.45516	-2.2	0.3333	0.3241	-2.8
79	GaAs_5kV	38	Ga	0.4820	JEOL8500f-Bruker2019	7,547,321	GaP	1.063	0.5055	±0.0030	4.9	0.4754	-1.4	0.5000	0.4933	-1.3
80	GaAs_5kV	38	As	0.5180	JEOL8500f-Bruker2019	7,547,321	As	1.063	0.5579	±0.0242	7.7	0.5246	1.3	0.5000	0.5067	1.3
81	GaP_5keV	39	P	0.3076	JEOL8500f-Bruker2019	6,615,542	Ca5(PO4)3F	0.9549	0.2876	±0.0023	-6.5	0.3012	-2.1	0.5000	0.4924	-1.5
82	GaP_5keV	39	Ga	0.6924	JEOL8500f-Bruker2019	6,615,542	GaAS	0.9549	0.6673	±0.0043	-3.6	0.6988	0.92	0.5000	0.5076	1.5
83	GaSb_5keV	40	Ga	0.3641	JEOL8500f-Bruker2019	5,581,455	GaP	1.0299	0.3720	±0.0177	2.2	0.3612	-0.80	0.5000	0.4968	-0.64
84	GaSb_5keV	40	Sb	0.6359	JEOL8500f-Bruker2019	5,581,455	Sb	1.0299	0.6579	±0.0054	3.5	0.6388	0.46	0.5000	0.5032	0.64
85	GeO2_5keV	41	O	0.3058	JEOL8500f-Bruker2019	6,110,745	SiO2	0.9395	0.2815	±0.0165	-7.9	0.2996	-2.0	0.6667	0.6601	-1.0
86	GeO2_5keV	41	Ge	0.6942	JEOL8500f-Bruker2019	6,110,745	Ge	0.9395	0.6581	±0.0165	-5.2	0.7004	0.89	0.3333	0.3399	2.0
87	GeTe_5kV	42	Ge	0.3628	JEOL8500f-Bruker2019	5,500,042	Ge	0.9312	0.3380	±0.0202	-6.8	0.3629	0.03	0.5000	0.5002	0.04
88	GeTe_5kV	42	Te	0.6372	JEOL8500f-Bruker2019	5,500,042	Te	0.9312	0.5933	±0.0086	-6.9	0.6371	-0.02	0.5000	0.4998	-0.04
89	NiO_5keV	43	O	0.2142	JEOL8500f-Bruker2019	5,846,515	MgO	1.0128	0.2139	±0.0086	-0.14	0.2112	-1.4	0.5000	0.4954	-0.92
90	NiO_5keV	43	Ni	0.7858	JEOL8500f-Bruker2019	5,846,515	Ni	1.0128	0.7989	±0.0159	1.7	0.7888	0.38	0.5000	0.5046	0.92
91	PbSe_5kV	44	Se	0.2759	JEOL8500f-Bruker2019	6,608,981	Se	0.9944	0.2760	±0.0029	0.04	0.2775	0.58	0.5000	0.5020	0.4
92	PbSe_5kV	44	Pb	0.7241	JEOL8500f-Bruker2019	6,608,981	PbTe	0.9944	0.7184	±0.0026	-0.79	0.7225	-0.4	0.5000	0.4980	-0.4
93	PbTe_5kV	45	Te	0.3811	JEOL8500f-Bruker2019	5,606,222	Te	0.9923	0.3691	±0.0043	-3.1	0.3720	-2.4	0.5000	0.4903	-1.9
94	PbTe_5kV	45	Pb	0.6189	JEOL8500f-Bruker2019	5,606,222	PbSe	0.9923	0.6232	±0.0022	0.69	0.6280	1.5	0.5000	0.5097	1.9
95	SiO2_5kV	46	O	0.5326	JEOL8500f-Bruker2019	5,109,005	NiO	0.9431	0.5028	±0.0240	-5.6	0.5331	0.09	0.6667	0.6672	0.07
96	SiO2_5kV	46	Si	0.4674	JEOL8500f-Bruker2019	5,109,005	Si	0.9431	0.4403	±0.0011	-5.8	0.4669	-0.11	0.3333	0.3328	-0.15
97	SnSe_5kV	47	Se	0.3995	JEOL8500f-Bruker2019	5,447,221	Se	0.9991	0.4055	±0.0047	1.5	0.4059	1.6	0.5000	0.5067	1.3
98	SnSe_5kV	47	Sn	0.6005	JEOL8500f-Bruker2019	5,447,221	Sn	0.9991	0.5936	±0.0023	-1.1	0.5941	-1.1	0.5000	0.4933	-1.3
99	Fe2O3_5kV	48	O	0.3006	JEOL8500f-Bruker2019	4,963,883	MgO	1.0675	0.3256	±0.0103	8.3	0.3050	1.5	0.6000	0.6050	0.83
100	Fe2O3_5kV	48	Fe	0.6994	JEOL8500f-Bruker2019	4,963,883	Fe	1.0675	0.7419	±0.0370	6.1	0.6950	-0.63	0.4000	0.3950	-1.3
101	Co-4Al	49	Al	0.0407	JEOL8500f-Bruker2019	2,949,880	Al	1.057	0.0436	±0.0007	7.1	0.0412	1.2	0.0848	0.0858	1.2
102	Co-4Al	49	Co	0.9593	JEOL8500f-Bruker2019	2,949,880	Co	1.057	1.0130	±0.0018	5.6	0.9588	-0.05	0.9152	0.9142	-0.11
103	Co77-Cr23	50	Cr	0.2308	JEOL8500f-Bruker2019	2,665,639	Cr	1.152	0.2588	±0.0210	12.1	0.2245	-2.7	0.2538	0.2471	-2.6
104	Co77-Cr23	50	Co	0.7692	JEOL8500f-Bruker2019	2,665,639	Co	1.152	0.8937	±0.0347	16.2	0.7755	0.82	0.7462	0.7529	0.90
105	Co50-Ta50	51	Co	0.5065	JEOL8500f-Bruker2019	3,323,134	WCo alloy	1.027	0.5077	±0.0112	0.24	0.4942	-2.4	0.7591	0.7500	-1.2
106	Co50-Ta50	51	Ta	0.4936	JEOL8500f-Bruker2019	3,323,134	Ta	1.027	0.5195	±0.0056	5.3	0.5058	2.5	0.2409	0.2500	3.8
107	Co50-W50	52	Co	0.5166	JEOL8500f-Bruker2019	3,256,689	CoTa alloy	1.006	0.5083	±0.0114	-1.6	0.5053	-2.2	0.7693	0.7611	-1.1
108	Co50-W50	52	W	0.4834	JEOL8500f-Bruker2019	3,256,689	W	1.006	0.4977	±0.0056	3.0	0.4947	2.3	0.2307	0.2389	3.6
109	Ni-8Al	53	Al	0.0773	JEOL8500f-Bruker2019	3,368,082	Cr	1.049	0.0805	±0.0014	4.1	0.0768	-0.65	0.1541	0.1532	-0.58
110	Ni-8Al	53	Ni	0.9227	JEOL8500f-Bruker2019	3,368,082	Ni	1.049	0.9681	±0.0028	4.9	0.9232	0.05	0.8459	0.8468	0.11
111	Ni75-Cr25	54	Cr	0.2513	JEOL8500f-Bruker2019	2,984,644	Cr	1.11	0.2704	±0.0228	7.6	0.2435	-3.1	0.2748	0.2666	-3.0
112	Ni75-Cr25	54	Ni	0.7487	JEOL8500f-Bruker2019	2,984,644	Ni	1.11	0.8399	±0.0214	12.2	0.7565	1.0	0.7252	0.7334	1.10
113	Ni80-Ta20	55	Ni	0.7829	JEOL8500f-Bruker2019	3,432,378	Ni	1.057	0.8330	±0.0134	6.4	0.7879	0.64	0.9175	0.9197	0.24
114	Ni80-Ta20	55	Ta	0.2171	JEOL8500f-Bruker2019	3,432,378	Ta	1.057	0.2242	±0.0038	3.3	0.2121	-2.3	0.0825	0.0803	-2.7
115	Ni-13W	56	Ni	0.8657	JEOL8500f-Bruker2019	3,399,203	Ni	1.029	0.8909	±0.0095	2.9	0.8655	-0.020	0.9528	0.9527	-0.01
116	Ni-13W	56	W	0.1343	JEOL8500f-Bruker2019	3,399,203	W	1.029	0.1384	±0.0027	3.1	0.1345	0.15	0.0472	0.0473	0.21
117	Cu75-Ni25	57	Ni	0.2497	JEOL8500f-Bruker2019	7,459,685	Ni	1.226	0.3173	±0.0151	27.1	0.2589	3.7	0.2656	0.2744	3.3
118	Cu75-Ni25	57	Cu	0.7474	JEOL8500f-Bruker2019	7,459,685	Cu	1.226	0.9083	±0.0318	21.5	0.7411	-0.84	0.7344	0.7256	-1.2
119	V3Si	58	Si	0.1552	JEOL8500f-Bruker2019	3,704,358	Si	1.048	0.1685	±0.0010	8.6	0.1609	3.6	0.2500	0.2580	3.2
120	V3Si	58	V	0.8448	JEOL8500f-Bruker2019	3,704,358	V	1.048	0.8790	±0.0263	6.2	0.8391	-0.67	0.7500	0.7420	-1.1
121	FeAl3	59	Al	0.5917	JEOL8500f-Bruker2019	5,829,447	Al	1.016	0.5952	±0.0037	0.59	0.5855	-1.0	0.7500	0.7452	-0.64
122	FeAl3	59	Fe	0.4213	JEOL8500f-Bruker2019	5,829,447	Fe2O3	1.016	0.4213	±0.0168	3.2	0.4145	1.5	0.2500	0.2548	1.9
123	Fe2Al5	60	Al	0.5471	JEOL8500f-Bruker2019	5,772,697	Al	1.009	0.5450	±0.0038	-0.38	0.5398	-1.3	0.7143	0.7083	-0.84
124	Fe2Al5	60	Fe	0.4529	JEOL8500f-Bruker2019	5,772,697	Fe2O3	1.009	0.4645	±0.0159	2.6	0.4602	1.6	0.2857	0.2917	2.1
125	ZnS_5keV	61	S	0.3290	JEOL8500f-Bruker2019	6,406,254	CuS	1.0214	0.3293	±0.0019	0.09	0.3224	-2.0	0.5000	0.4925	-1.5
126	ZnS_5keV	61	Zn	0.6710	JEOL8500f-Bruker2019	6,406,254	Zn	1.0214	0.6921	±0.0043	3.1	0.6776	0.98	0.5000	0.5075	1.5
127	Al2O3_5keV	62	O	0.4707	TESCAN_PulseTor	2,329,635	SiO2	1.076	0.4983	±0.0214	5.9	0.4631	-1.6	0.6000	0.5908	-1.2
128	Al2O3_5keV	62	Al	0.5293	TESCAN_PulseTor	2,329,635	Al	1.076	0.5777	±0.0020	9.1	0.5369	2.3	0.4000	0.4074	1.9
129	Anhydrite_5keV	63	S	0.2355	TESCAN_PulseTor	1,632,269	Cu2S	0.9678	0.2245	±0.0013	-4.7	0.2319	-1.5	0.1667	0.1646	-1.3
130	Anhydrite_5keV	63	Ca	0.2955	TESCAN_PulseTor	1,632,269	CaCO3	0.9678	0.2911	±0.0016	-1.1	0.3008	2.2	0.1667	0.1708	2.5
131	Arsenopyrite_5keV	64	S	0.1969	TESCAN_PulseTor	2,093,550	Pyrite	1.0194	0.1970	±0.0009	0.05	0.1933	-1.8	0.3333	0.3272	-1.8
132	Arsenopyrite_5keV	64	Fe	0.3430	TESCAN_PulseTor	2,093,550	Pyrite	1.0194	0.3633	±0.0085	5.9	0.3563	3.9	0.3333	0.3464	3.9
133	Arsenopyrite_5keV	64	As	0.4601	TESCAN_PulseTor	2,093,550	InAs	1.0194	0.4592	±0.0075	-0.20	0.4504	-2.1	0.3333	0.3264	-2.1
134	BaAl2Si2O8_celsian_5keV	65	Al	0.1437	TESCAN_PulseTor	2,029,779	Albite	1.0411	0.1412	±0.0025	-1.7	0.1357	-5.6	0.1538	0.1473	-4.2
135	BaAl2Si2O8_celsian_5keV	65	Si	0.1496	TESCAN_PulseTor	2,029,779	Albite	1.0411	0.1565	±0.0009	4.6	0.1503	0.47	0.1538	0.1568	2.0
136	BaAl2Si2O8_celsian_5keV	65	Ba	0.3658	TESCAN_PulseTor	2,029,779	Barite	1.0411	0.3936	±0.0062	7.6	0.3780	3.3	0.0769	0.8070	4.9
137	Chalcopyrite_5keV	66	S	0.3494	TESCAN_PulseTor	1,899,047	ZnS	0.9465	0.3246	±0.0020	-7.1	0.3429	-1.9	0.5000	0.4927	-1.5
138	Chalcopyrite_5keV	66	Fe	0.3043	TESCAN_PulseTor	1,899,047	Fe2P	0.9465	0.2921	±0.0240	-4.0	0.3086	1.4	0.2500	0.2546	1.8
139	Chalcopyrite_5keV	66	Cu	0.3463	TESCAN_PulseTor	1,899,047	Cu	0.9465	0.3298	±0.0222	-4.8	0.3485	0.64	0.2500	0.2527	1.1
140	Cinnabar_5keV	67	S	0.1378	TESCAN_PulseTor	2,158,611	Cu2S	0.9649	0.1376	±0.0046	-0.15	0.1426	3.5	0.5000	0.5098	2.0
141	Cinnabar_5keV	67	Hg	0.8622	TESCAN_PulseTor	2,158,611	HgTe	0.9649	0.8273	±0.0032	-4.0	0.8574	-0.56	0.5000	0.4903	-2.0
142	Cryolite_5keV	68	F	0.5430	TESCAN_PulseTor	2,546,579	GdF3	1.0366	0.5504	±0.0113	1.4	0.5310	-2.2	0.6000	0.5880	-2.0
143	Cryolite_5keV	68	Na	0.3285	TESCAN_PulseTor	2,546,579	Albite	1.0366	0.3551	±0.0059	8.1	0.3426	4.3	0.3000	0.3135	-4.5
144	Cryolite_5keV	68	Al	0.1285	TESCAN_PulseTor	2,546,579	Al2O3	1.0366	0.1311	±0.0011	2.0	0.1264	-1.6	0.1000	0.0986	-1.4
145	Cu2S_5keV	69	S	0.2015	TESCAN_PulseTor	12,932,635	Pyrite	1.0852	0.2208	±0.0012	9.6	0.2035	0.99	0.3333	0.3361	0.8
146	Cu2S_5keV	69	Cu	0.7985	TESCAN_PulseTor	12,932,635	Cu	1.0852	0.8644	±0.0127	8.3	0.7965	-0.25	0.6667	0.6639	-0.42
147	Diopside_5keV	70	Mg	0.1122	TESCAN_PulseTor	1,804,254	MgAl2O4	0.9931	0.1069	±0.0003	-4.7	0.1077	-4.0	0.1000	0.0959	-4.1
148	Diopside_5keV	70	Si	0.2594	TESCAN_PulseTor	1,804,254	Quartz	0.9931	0.2608	±0.0005	0.54	0.2626	1.2	0.2000	0.2025	1.3
149	Diopside_5keV	70	Ca	0.1851	TESCAN_PulseTor	1,804,254	Calcite	0.9931	0.1843	±0.0012	0.43	0.1856	0.27	0.1000	0.1003	0.30
150	Galena_5keV	71	S	0.1340	TESCAN_PulseTor	2,096,760	FeS	0.8386	0.1103	±0.0007	-17.7	0.1315	-1.9	0.5000	0.4946	-1.1
151	Galena_5keV	71	Pb	0.8660	TESCAN_PulseTor	2,096,760	PbSe	0.8386	0.7283	±0.0032	-15.9	0.8685	0.29	0.5000	0.5054	1.1
152	Jadeite_5keV	72	Na	0.1137	TESCAN_PulseTor	2,160,059	Albite	1.0079	0.1149	±0.0002	1.1	0.1140	0.26	0.1000	0.1002	0.20
153	Jadeite_5keV	72	Al	0.1335	TESCAN_PulseTor	2,160,059	Albite	1.0079	0.1326	±0.0002	-0.67	0.1315	-1.5	0.1000	0.0985	-1.5
154	Jadeite_5keV	72	Si	0.2779	TESCAN_PulseTor	2,160,059	Albite	1.0079	0.2816	±0.0003	1.3	0.2794	0.54	0.2000	0.2011	0.55
155	Kyanite_5keV	73	Al	0.3330	TESCAN_PulseTor	2,166,158	Albite	0.9987	0.3298	±0.0007	-0.96	0.3302	-0.84	0.2500	0.2479	-0.84
156	Kyanite_5keV	73	Si	0.1733	TESCAN_PulseTor	2,166,158	Albite	0.9987	0.1754	±0.0007	1.2	0.1756	1.3	0.1250	0.1267	1.40
157	Magnetite_Fe3O4_5keV	74	O	0.2764	TESCAN_PulseTor	9,294,053	Hematite	0.9473	0.2661	±0.0004	-3.7	0.2809	1.6	0.5714	0.5769	0.96
158	Magnetite_Fe3O4_5keV	74	Fe	0.7236	TESCAN_PulseTor	9,294,053	Hematite	0.9473	0.6812	±0.0026	-5.9	0.7191	-0.62	0.4286	0.4231	-1.3
159	Hematite_24_5keV	75	O	0.3006	TESCAN_PulseTor	1,900,529	Magnetite	1.0177	0.2918	±0.0004	-2.9	0.2867	-4.6	0.6000	0.5839	-2.7
160	Hematite_24_5keV	75	Fe	0.6994	TESCAN_PulseTor	1,900,529	Magnetite	1.0177	0.7259	±0.0016	3.8	0.7133	2.0	0.4000	0.4161	4.0
161	MoS2_Molybdenite	76	S	0.4006	TESCAN_PulseTor	7,827,290	FeS2	0.8043	0.3223	±0.0014	-19.5	0.4007	0.03	0.6667	0.6667	0.0
162	MoS2_Molybdenite	76	Mo	0.5994	TESCAN_PulseTor	7,827,290	Mo	0.8043	0.4820	±0.0025	-19.6	0.5993	-0.02	0.3333	0.3333	0.0
163	Pyrite_43_5keV	77	S	0.5345	TESCAN_PulseTor	8,013,382	Cu2S	0.9142	0.4873	±0.0032	-8.8	0.5330	-0.28	0.6667	0.6653	-0.21
164	Pyrite_43_5keV	77	Fe	0.4655	TESCAN_PulseTor	8,013,382	FeSi2	0.9142	0.4269	±0.0110	-8.3	0.4670	0.32	0.3333	0.3347	0.42
165	Scheelite_CaWO4_5keV	78	Ca	0.1392	TESCAN_PulseTor	10,234,029	CaF2	0.9302	0.1330	±0.0010	-4.4	0.1429	2.7	0.1667	0.1704	2.2
166	Scheelite_CaWO4_5keV	78	W	0.6385	TESCAN_PulseTor	10,234,029	W	0.9302	0.5901	±0.0051	-7.6	0.6344	-0.64	0.1667	0.1648	-1.1
167	Si3N4_5keV	79	N	0.3994	TESCAN_PulseTor	1,568,369	AlN	0.9805	0.3855	±0.0667	-3.5	0.3932	-1.5	0.5714	0.5650	-1.1
168	Si3N4_5keV	79	Si	0.6006	TESCAN_PulseTor	1,568,369	SiO2	0.9805	0.5950	±0.0017	-0.93	0.6068	1.0	0.4286	0.4350	1.5
169	SiC_5keV	80	C	0.2995	TESCAN_PulseTor	1,498,148	C	1.0299	0.3054	±0.1112	2.0	0.2965	-1.0	0.5000	0.4964	-0.72
170	SiC_5keV	80	Si	0.7005	TESCAN_PulseTor	1,498,148	Si	1.0299	0.7245	±0.0007	3.4	0.7035	0.43	0.5000	0.5036	0.72
171	TiC_5keV	81	C	0.2006	TESCAN_PulseTor	3,763,008	C	0.9865	0.2049	±0.0316	2.1	0.2077	3.5	0.5000	0.5109	2.2
172	TiC_5keV	81	Ti	0.7994	TESCAN_PulseTor	3,763,008	Ti	0.9865	0.7816	±0.0552	-2.2	0.7923	-0.89	0.5000	0.4891	-2.2
173	Willemite_Zn2SiO4_5keV	82	Si	0.1260	TESCAN_PulseTor	12,259,857	SiO2	0.8786	0.1105	±0.0012	-12.3	0.1258	-0.16	0.1429	0.1427	-0.14
174	Willemite_Zn2SiO4_5keV	82	Zn	0.5869	TESCAN_PulseTor	12,259,857	ZnS	0.8786	0.5159	±0.0047	-12.1	0.5872	0.05	0.2857	0.2860	0.11
175	Wollastonite_CaSiO3_5keV	83	Si	0.2418	TESCAN_PulseTor	7,690,016	Quartz	0.9847	0.2382	±0.0005	-1.5	0.2420	0.08	0.2000	0.2001	0.05
176	Wollastonite_CaSiO3_5keV	83	Ca	0.3450	TESCAN_PulseTor	7,690,016	Calcite	0.9847	0.3395	±0.0018	-1.6	0.3448	-0.06	0.2000	0.1998	-0.10
177	Zircon_ZrSiO4_5keV	84	Si	0.1532	TESCAN_PulseTor	9,454,032	Quartz	0.9359	0.1468	±0.0005	-4.2	0.1568	2.3	0.1667	0.1696	1.7
178	Zircon_ZrSiO4_5keV	84	Zr	0.4930	TESCAN_PulseTor	9,454,032	Zr	0.9359	0.4604	±0.0016	-6.6	0.4919	-0.22	0.1667	0.1638	-1.7
179	ZnS_5keV	85	S	0.3290	TESCAN_PulseTor	2,332,147	Cu2S	0.9539	0.3145	±0.0025	-4.4	0.3297	0.21	0.5000	0.5008	0.16
180	ZnS_5keV	85	Zn	0.6710	TESCAN_PulseTor	2,332,147	Zn	0.9539	0.6394	±0.0048	-4.7	0.6703	-0.10	0.5000	0.4992	-0.16
181	SiO2_5keV	86	O	0.5326	TESCAN_PulseTor	2,030,769	MgO	0.9985	0.5222	±0.0232	-2.0	0.5230	-1.8	0.6667	0.6581	-1.3
182	SiO2_5keV	86	Si	0.4674	TESCAN_PulseTor	2,030,769	Si	0.9985	0.4763	±0.0013	1.9	0.4770	2.1	0.3333	0.3419	2.6
183	SrSO4_Celestite_5keV	87	S	0.1746	TESCAN_PulseTor	1,813,422	Pyrite	1.0334	0.1853	±0.0031	6.1	0.1793	2.7	0.1667	0.1693	1.6
184	SrSO4_Celestite_5keV	87	Sr	0.4770	TESCAN_PulseTor	1,813,422	SrTiO3	1.0334	0.4825	±0.0010	1.2	0.4669	-2.1	0.1667	0.1613	-3.2
185	SRM470_K412_5keV	88	O	0.4276	TESCAN_PulseTor	1,993,359	MgO	0.9873	0.4164	±0.0152	-2.6	0.4217	-1.4	0.5940	0.5854	-1.4
186	SRM470_K412_5keV	88	Mg	0.1166	TESCAN_PulseTor	1,993,359	MgO	0.9873	0.1155	±0.0004	-0.98	0.1169	0.26	0.1066	0.1068	0.21
187	SRM470_K412_5keV	88	Al	0.0491	TESCAN_PulseTor	1,993,359	Al2O3	0.9873	0.0479	±0.0003	-2.4	0.0485	-1.2	0.0404	0.0400	-1.0
188	SRM470_K412_5keV	88	Si	0.2120	TESCAN_PulseTor	1,993,359	SiO2	0.9873	0.2151	±0.0006	1.4	0.2178	2.7	0.1678	0.1722	2.6
189	SRM470_K412_5keV	88	Ca	0.1090	TESCAN_PulseTor	1,993,359	CaF2	0.9873	0.1133	±0.0019	4.0	0.1148	5.3	0.0604	0.0636	5.3
190	SRM470_K412_5keV	88	Fe	0.0774	TESCAN_PulseTor	1,993,359	FeSi2	0.9873	0.0793	±0.0065	2.5	0.0803	3.8	0.0308	0.0320	3.8
191	Fe2P_5keV	89	P	0.2171	TESCAN_PulseTor	1,824,744	Fluorapatite	1.097	0.2288	±0.0014	5.4	0.2086	-3.9	0.3333	0.3222	-3.3
192	Fe2P_5keV	89	Fe	0.7829	TESCAN_PulseTor	1,824,744	Fe2O3	1.097	0.8680	±0.0242	10.9	0.7914	1.1	0.6667	0.6778	1.7
193	Scheelite_CaWO4_5keV	90	Ca	0.1392	TESCAN_EDAX	5,594,523	CaF2	1.0459	0.1390	±0.0008	-0.14	0.1329	-4.5	0.1667	0.1604	-3.8
194	Scheelite_CaWO4_5keV	90	W	0.6385	TESCAN_EDAX	5,594,523	W	1.0459	0.6751	±0.0054	5.7	0.6455	1.1	0.1667	0.1698	1.9
195	Cr3C2_5keV	91	C	0.1334	TESCAN_EDAX	2,281,143	C	0.9715	0.1254	±0.0266	-6.0	0.1294	-3.0	0.4000	0.3914	-2.2
196	Cr3C2_5keV	91	Cr	0.8666	TESCAN_EDAX	2,281,143	Cr	0.9715	0.8449	±0.0215	-2.5	0.8706	0.47	0.6000	0.6086	1.4
197	Cr2N_5keV	92	N	0.1187	TESCAN_EDAX	2,402,564	Si3N4	0.9299	0.1201	±0.0205	1.2	0.1292	8.8	0.3333	0.3551	6.5
198	Cr2N_5keV	92	Cr	0.8813	TESCAN_EDAX	2,402,564	Cr	0.9299	0.8097	±0.0270	-8.1	0.8708	-1.2	0.6667	0.6449	-3.3
199	Fe3N_5keV	93	N	0.0772	TESCAN_EDAX	5,009,859	GaN	0.9988	0.0789	±0.0113	2.2	0.0788	2.0	0.2500	0.2534	1.4
200	Fe3N_5keV	93	Fe	0.9228	TESCAN_EDAX	5,009,859	Fe	0.9988	0.9199	±0.0114	-0.32	0.9212	-0.17	0.7500	0.7467	-0.46
201	Magnetite_Fe3O4_5keV	94	O	0.2764	TESCAN_EDAX	5,731,541	MgAl2O4	0.883	0.2444	±0.0106	-11.6	0.2768	0.14	0.5714	0.5744	0.53
202	Magnetite_Fe3O4_5keV	94	Fe	0.7236	TESCAN_EDAX	5,731,541	Fe	0.883	0.6386	±0.0409	-11.7	0.7232	-0.06	0.4286	0.4256	-0.70
203	Si3N4_5keV	95	N	0.3994	TESCAN_EDAX	2,600,016	GaN	0.9445	0.3854	±0.0804	-3.5	0.4078	2.1	0.5714	0.5719	0.09
204	Si3N4_5keV	95	Si	0.6006	TESCAN_EDAX	2,600,016	Si	0.9445	0.5591	±0.0011	-6.9	0.5922	-1.4	0.4286	0.4281	-0.12
205	SiC_5keV	96	C	0.2995	TESCAN_EDAX	2,096,108	C	0.9801	0.2977	±0.1265	-0.60	0.3038	1.4	0.5000	0.4962	-0.77
206	SiC_5keV	96	Si	0.7005	TESCAN_EDAX	2,096,108	Si	0.9801	0.6824	±0.0009	-2.6	0.6962	-0.61	0.5000	0.5038	0.77
207	BN_5keV	97	B	0.4356	TESCAN_EDAX	2,113,824	B	0.9919	0.4100	±0.0955	-5.9	0.4133	-5.1	0.5000	0.4772	-4.6
208	BN_5keV	97	N	0.5644	TESCAN_EDAX	2,113,824	Si3N4	0.9919	0.5819	±0.1039	3.1	0.5867	4.0	0.5000	0.5228	4.6
209	Dolomite_CaMgC2O6	98	C	0.1303	TESCAN_EDAX	5,211,942	C	1.004	0.1372	±0.0160	5.3	0.1367	4.9	0.2000	0.2099	5.0
210	Dolomite_CaMgC2O6	98	O	0.5206	TESCAN_EDAX	5,211,942	MgCaSi2O6	1.004	0.5113	±0.0128	-1.8	0.5092	-2.2	0.6000	0.5870	-2.2
211	Dolomite_CaMgC2O6	98	Mg	0.1318	TESCAN_EDAX	5,211,942	MgCaSi2O6	1.004	0.1347	±0.0005	2.2	0.1342	1.8	0.1000	0.1018	1.8
212	Dolomite_CaMgC2O6	98	Ca	0.2173	TESCAN_EDAX	5,211,942	MgCaSi2O6	1.004	0.2208	±0.0014	1.6	0.2199	1.2	0.1000	0.1012	1.2
213	Rhodonite_MnSiO3	99	Si	0.2144	TESCAN_EDAX	5,655,480	Quartz	1.009	0.2155	±0.0009	0.51	0.2136	-0.37	0.2000	0.1995	-0.25
214	Rhodonite_MnSiO3	99	Mn	0.4193	TESCAN_EDAX	5,655,480	Mn	1.009	0.4243	±0.0479	1.2	0.4205	0.29	0.2000	0.2008	0.40
215	Pentlandite_Fe4Ni5S8_5keV	100	S	0.3317	TESCAN_EDAX	5,001,847	Pyrite	1.0007	0.3350	±0.0010	0.99	0.3227	0.30	0.4706	0.4719	0.28
216	Pentlandite_Fe4Ni5S8_5keV	100	Fe	0.2888	TESCAN_EDAX	5,001,847	Fe	1.0007	0.2781	±0.0350	-3.7	0.2761	-4.4	0.2353	0.2249	-4.4
217	Pentlandite_Fe4Ni5S8_5keV	100	Ni	0.3795	TESCAN_EDAX	5,001,847	Ni	1.0007	0.3939	±0.0313	3.8	0.3912	3.1	0.2941	0.3031	3.1
218	MgAl2O4_5keV	101	O	0.4498	TESCAN_EDAX	6,467,268	Kyanite	0.9879	0.4403	±0.0074	-2.1	0.4457	-0.91	0.5714	0.5674	-0.70
219	MgAl2O4_5keV	101	Mg	0.1708	TESCAN_EDAX	6,467,268	Dolomite	0.9879	0.1691	±0.0006	-1.0	0.1712	0.23	0.1429	0.1434	0.35
220	MgAl2O4_5keV	101	Al	0.3793	TESCAN_EDAX	6,467,268	Kyanite	0.9879	0.3785	±0.0015	-0.21	0.3831	1.0	0.2875	0.2892	1.2
221	Fluorapatite_Ca5P3O12F	102	O	0.3807	TESCAN_EDAX	4,318,131	CaSO4	1.031	0.3956	±0.0127	3.9	0.3839	0.84	0.5714	0.5729	0.26
222	Fluorapatite_Ca5P3O12F	102	P	0.1843	TESCAN_EDAX	4,318,131	CeP5O14	1.031	0.1945	±0.0007	5.5	0.1888	2.4	0.1429	0.1455	1.8
223	Fluorapatite_Ca5P3O12F	102	Ca	0.3974	TESCAN_EDAX	4,318,131	CaSO4	1.031	0.3985	±0.0017	0.28	0.3867	-2.7	0.2381	0.2304	-3.2
224	Albite_NaAlSi3O8_5keV	103	O	0.4881	TESCAN_EDAX	5,741,936	Al2SiO5	0.9769	0.4781	±0.0089	-2.0	0.4894	0.27	0.6154	0.6164	0.16
225	Albite_NaAlSi3O8_5keV	103	Na	0.0877	TESCAN_EDAX	5,741,936	NaAlSi2O6	0.9769	0.0865	±0.0001	-1.4	0.0885	0.91	0.0769	0.0776	0.91
226	Albite_NaAlSi3O8_5keV	103	Al	0.1029	TESCAN_EDAX	5,741,936	Al2SiO5	0.9769	0.1027	±0.0002	-0.19	0.1052	2.2	0.0769	0.0786	2.2
227	Albite_NaAlSi3O8_5keV	103	Si	0.3213	TESCAN_EDAX	5,741,936	Al2SiO5	0.9769	0.3096	±0.0013	-3.6	0.3170	-1.3	0.2308	0.2274	-1.5
228	Calcite_CaCO3_5keV	104	C	0.1200	TESCAN_EDAX	4,380,573	C	1.013	0.1174	±0.0129	-2.2	0.1158	-3.5	0.2000	0.1932	-3.4
229	Calcite_CaCO3_5keV	104	O	0.4796	TESCAN_EDAX	4,380,573	CaSO4	1.013	0.4917	±0.0159	2.5	0.4852	1.2	0.6000	0.6074	1.2
230	Calcite_CaCO3_5keV	104	Ca	0.4004	TESCAN_EDAX	4,380,573	CaSO4	1.013	0.4044	±0.0017	1.0	0.3990	-0.35	0.2000	0.1994	-0.30
231	Arsenopyrite_FeAsS_5keV	105	S	0.1969	TESCAN_EDAX	5,247,349	FeS2	1.044	0.1989	±0.0009	1.0	0.1905	-3.3	0.3333	0.3238	-2.9
232	Arsenopyrite_FeAsS_5keV	105	Fe	0.3430	TESCAN_EDAX	5,247,349	FeS2	1.044	0.3679	±0.0092	7.3	0.3524	2.7	0.3333	0.3438	3.2
233	Arsenopyrite_FeAsS_5keV	105	As	0.4601	TESCAN_EDAX	5,247,349	As2O3	1.044	0.4772	±0.0048	3.7	0.4571	-0.65	0.3333	0.3324	-0.27
234	Galena_PbS_5keV	106	S	0.1340	TESCAN_EDAX	5,108,901	CaSO4	1.017	0.1354	±0.0009	1.0	0.1332	-0.60	0.5000	0.4981	-0.38
235	Galena_PbS_5keV	106	Pb	0.8660	TESCAN_EDAX	5,108,901	PbCrO4	1.017	0.8815	±0.0026	1.7	0.8668	0.09	0.5000	0.5019	0.38
236	B4C_5keV	107	B	0.7826	TESCAN_EDAX	1,040,373	B	1.084	0.8582	±0.0493	9.7	0.7919	1.2	0.8000	0.8087	1.1
237	B4C_5keV	107	C	0.2174	TESCAN_EDAX	1,040,373	C	1.084	0.2255	±0.0913	3.7	0.2081	-4.3	0.2000	0.1913	-4.4
238	SRM1871_K456_O-Si-Pb_5keV	108	O	0.2043	TESCAN_EDAX	5,177,155	SiO2	1.077	0.2265	±0.0162	10.9	0.2099	2.7	0.6154	0.6269	1.9
239	SRM1871_K456_O-Si-Pb_5keV	108	Si	0.1345	TESCAN_EDAX	5,177,155	SiO2	1.077	0.1401	±0.0008	4.2	0.1299	-3.4	0.2308	0.2209	-4.3
240	SRM1871_K456_O-Si-Pb_5keV	108	Pb	0.6612	TESCAN_EDAX	5,177,155	PbTe	1.077	0.7123	±0.0034	7.7	0.6602	-0.15	0.1538	0.1522	-1.0
241	SRM1872_K453_O-Ge-Pb_5keV	109	O	0.1673	TESCAN_EDAX	5,741,186	MgO	1.099	0.1901	±0.0123	13.7	0.1730	3.4	0.6156	0.6249	1.5
242	SRM1872_K453_O-Ge-Pb_5keV	109	Ge	0.2843	TESCAN_EDAX	5,741,186	Ge	1.099	0.3071	±0.0183	8.0	0.2793	-1.8	0.2304	0.2223	-3.5
243	SRM1872_K453_O-Ge-Pb_5keV	109	Pb	0.5421	TESCAN_EDAX	5,741,186	PbTe	1.099	0.6022	±0.0033	11.1	0.5477	1.0	0.1540	0.1528	-0.78
244	SRM1873_K458_O-Si-Zn-Ba_5keV	110	O	0.3186	TESCAN_EDAX	5,292,415	SiO2	1.056	0.3394	±0.0086	6.5	0.3214	0.88	0.6297	0.6318	0.3
245	SRM1873_K458_O-Si-Zn-Ba_5keV	110	Si	0.2305	TESCAN_EDAX	5,292,415	SiO2	1.056	0.2432	±0.0016	5.5	0.2304	-0.01	0.2595	0.2579	-0.62
246	SRM1873_K458_O-Si-Zn-Ba_5keV	110	Zn	0.0301	TESCAN_EDAX	5,292,415	Zn2SiO4	1.056	0.0322	±0.0008	7.0	0.0305	1.3	0.0146	0.0147	0.68
247	SRM1873_K458_O-Si-Zn-Ba_5keV	110	Ba	0.4179	TESCAN_EDAX	5,292,415	BaSO4	1.056	0.4283	±0.0084	2.2	0.4176	-0.07	0.0962	0.0956	-0.62
248	SRM1875-K496_O-Mg-Al-P_5keV	111	O	0.5390	TESCAN_EDAX	5,376,238	MgO	1.108	0.5953	±0.0222	10.4	0.5373	-0.32	0.6810	0.6792	-0.26
249	SRM1875-K496_O-Mg-Al-P_5keV	111	Mg	0.0665	TESCAN_EDAX	5,376,238	MgO	1.108	0.0770	±0.0003	15.8	0.0695	4.5	0.0553	0.0579	4.7
250	SRM1875-K496_O-Mg-Al-P_5keV	111	Al	0.0647	TESCAN_EDAX	5,376,238	Al2O3	1.108	0.0699	±0.0003	8.0	0.0631	-2.5	0.0485	0.0473	-2.5
251	SRM1875-K496_O-Mg-Al-P_5keV	111	P	0.3298	TESCAN_EDAX	5,376,238	GaP	1.108	0.3658	±0.0030	10.9	0.3301	0.09	0.2152	0.2156	0.19
252	SRM470_K411_O-Mg-Si-Ca-Fe_5keV	112	O	0.4237	TESCAN_EDAX	1,146,015	MgO	0.9745	0.4154	±0.0174	-2.0	0.4263	0.61	0.6029	0.6005	-0.40
253	SRM470_K411_O-Mg-Si-Ca-Fe_5keV	112	Mg	0.0885	TESCAN_EDAX	1,146,015	MgO	0.9745	0.0870	±0.0005	-1.7	0.0893	0.90	0.0829	0.0828	-0.12
254	SRM470_K411_O-Mg-Si-Ca-Fe_5keV	112	Si	0.2538	TESCAN_EDAX	1,146,015	SiO2	0.9745	0.2513	±0.0009	-0.99	0.2579	1.6	0.2058	0.2069	0.53
255	SRM470_K411_O-Mg-Si-Ca-Fe_5keV	112	Ca	0.1106	TESCAN_EDAX	1,146,015	CaF2	0.9745	0.1126	±0.0033	1.8	0.1155	4.4	0.0628	0.0650	3.4
256	SRM470_K411_O-Mg-Si-Ca-Fe_5keV	112	Fe	0.1121	TESCAN_EDAX	1,146,015	K412	0.9745	0.1083	±0.0011	-3.4	0.1111	-0.89	0.0457	0.0448	-2.0
257	SRM470_K412_O-Mg-Al-Si-Ca-Fe_5keV	113	O	0.4276	TESCAN_EDAX	1,150,087	MgO	0.9572	0.4085	±0.0163	-4.5	0.4268	-0.19	0.5940	0.5903	-0.62
258	SRM470_K412_O-Mg-Al-Si-Ca-Fe_5keV	113	Mg	0.1166	TESCAN_EDAX	1,150,087	MgO	0.9572	0.1125	±0.0163	-3.5	0.1176	0.86	0.1066	0.1070	0.37
259	SRM470_K412_O-Mg-Al-Si-Ca-Fe_5keV	113	Al	0.0491	TESCAN_EDAX	1,150,087	Al2O3	0.9572	0.0475	±0.0004	-3.3	0.0496	1.00	0.0404	0.0407	0.64
260	SRM470_K412_O-Mg-Al-Si-Ca-Fe_5keV	113	Si	0.2120	TESCAN_EDAX	1,150,087	SiO2	0.9572	0.2058	±0.0008	-2.9	0.2151	1.50	0.1678	0.1694	0.95
261	SRM470_K412_O-Mg-Al-Si-Ca-Fe_5keV	113	Ca	0.1090	TESCAN_EDAX	1,150,087	CaF2	0.9572	0.1035	±0.0032	-5.0	0.1081	-0.83	0.0604	0.0597	-1.2
262	SRM470_K412_O-Mg-Al-Si-Ca-Fe_5keV	113	Fe	0.0774	TESCAN_EDAX	1,150,087	K411	0.9572	0.0793	±0.0009	2.4	0.0829	7.1	0.0308	0.0328	6.6

All raw materials were materialographically prepared in our laboratory following appropriate mounting, grinding and polishing procedures. Materials in the form of fine powders were mounted in conducting epoxy. Non-conductive materials were coated with a thin (approximately 10 nm) carbon layer applied by thermal evaporation. Homogeneity of the materials was confirmed by point beam sampling.

Standards for analysis consisted of a suite of pure elements, e.g., B, C, Si, Ti, Cr, Fe, Ni, Cu, Zn, Mo, etc., and for those pure elements that are incompatible with the instrument vacuum requirements or which are unstable under electron beam bombardment, stoichiometric compounds, e.g., MgO, FeS_2_, KCl, etc., were utilized. Non-conductive standards were coated with a thin (approximately 10 nm) carbon layer applied by thermal evaporation.

### Selection of valid measurements

**Figure 1:**
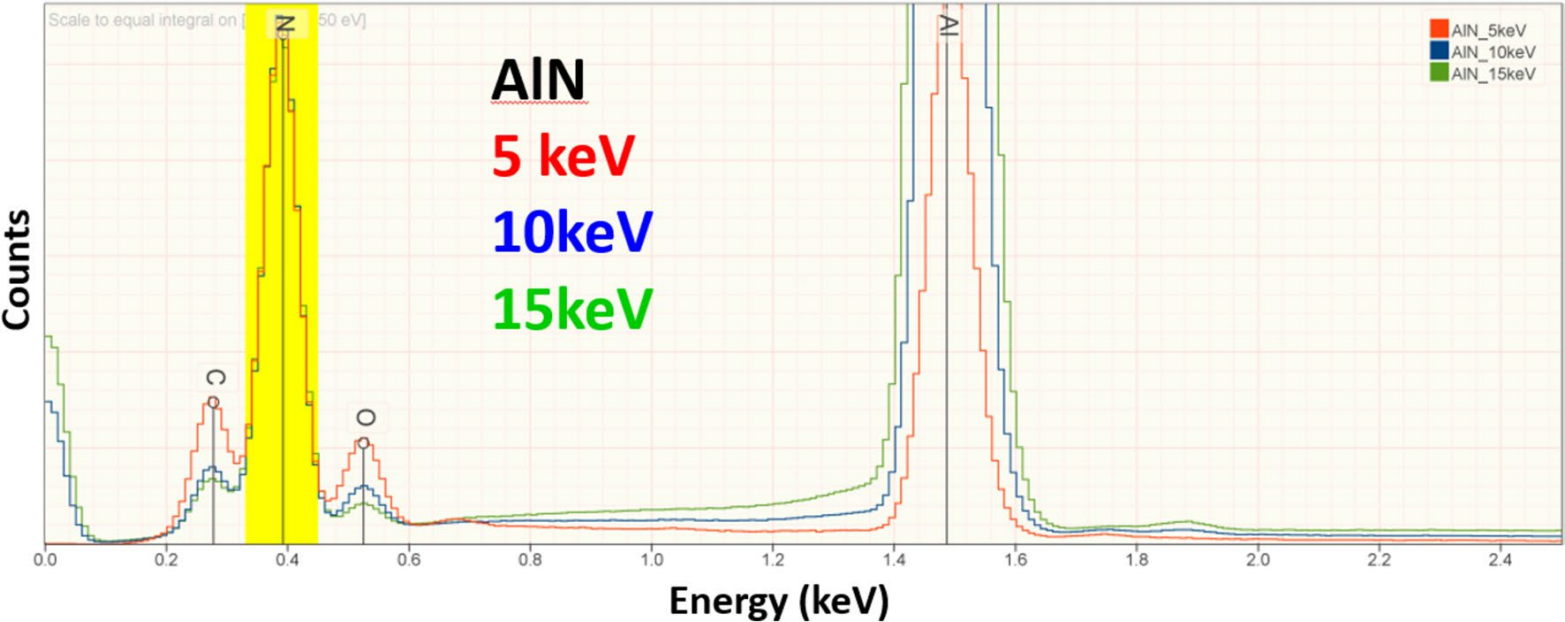
EDS spectra of AlN recorded at E_0_ = 15 keV, 10 keV and 5 keV. Scaling to the N K peak (yellow) shows the increasing intensity of the C and O peaks as the beam energy is reduced.

**Figure 2:**
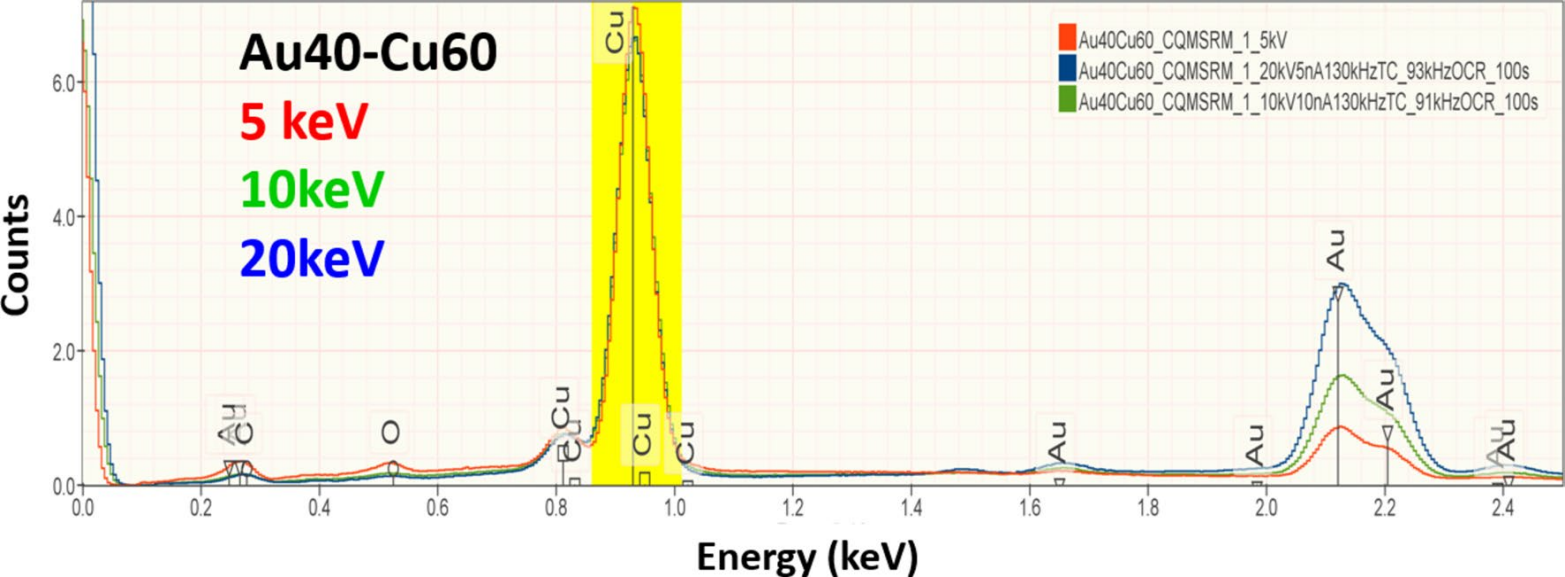
EDS spectra of NIST SRM 482 (Au40-Cu60) recorded at E_0_ = 20 keV, 10 keV and 5 keV. Scaling to the Cu L family peak (yellow) shows only a small increase in the relative intensity of the C and O peaks as the beam energy is reduced.

As described in a previous publication [2], microanalysis performed at low beam energy is likely to encounter surface layers that naturally form on materials as a result of oxidation, sulfidation, etc., upon exposure to the atmosphere. Since such a layer effectively creates a composite material, this situation can significantly compromise the accuracy of the analysis. For this study, the EDS spectrum was examined as a function of beam energy to assess the possible existence of such surface layers. Figure 1 shows an example of an AlN specimen which was excluded from this study because the presence of significant C and O became apparent as the beam energy was reduced. An example of a material deemed acceptable for this study is shown in Figure 2, where EDS spectra of NIST SRM 482 (Au40-Cu60) recorded at E_0_ = 20 keV, 10 keV, and 5 keV displayed with scaling to the Cu L family peak (yellow band) reveals only a small increase in the relative intensity of the C and O peaks as the beam energy is reduced.

### Measurement instrumentation

Two different electron beam platforms were utilized and each was fitted with two different EDS spectrometers during the extended period of data collection for this study:

1. A JEOL 8500F electron probe microanalyzer (EPMA) equipped with (1) Bruker four-detector array of silicon drift detector EDS (four detectors with a total of 40 mm^2^ active area at 72 mm source-to-detector distance) or (2) a Bruker single silicon drift detector of 30 mm^2^ active area at 72 mm source-to-detector distance.
2. A TESCAN MIRA3 LMU scanning electron microscope equipped (1) with four 30 mm^2^ PulseTor silicon drift detectors (two at 34 mm and two at 38 mm source-to-detector distance) or (2) four EDAX silicon drift detectors controlled by the SEMantics extension to NIST DTSA-II [7].

### Spectrum processing

EDS spectra were collected using the particular vendor software and then subsequently exported using the ISO/EMSA Spectrum File Format (Microscopy Society of America, Reston, VA; ISO 22029:2012) to the open source EDS software platform NIST DTSA-II for all spectral processing calculations [3]. The NIST DTSA-II software is available free at the NIST website (search “DTSA” at www.nist.gov): https://www.nist.gov/services-resources/software/nist-dtsa-ii and the source is available from https://github.com/usnistgov/DTSA-II and https://github.com/usnistgov/epq.

All results were obtained by following the standards-based *k-ratio* quantitative analysis protocol in which the characteristic X-ray intensity, ${I}_{X}$, for each element, $i$, (corrected for any peak interferences and for the X-ray continuum background) is ratioed to the intensity measured on a standard containing that element, ${C}_{i,stan}$, under carefully defined measurement conditions (e.g., monitoring of the beam current throughout each measurement sequence of challenge materials and appropriate standards to ensure a constant dose was achieved):

1 $$k_{i} = I_{x,sample} /I_{x,standard}$$

The suite of k-ratios is then converted into a suite of mass concentrations by calculating a series of matrix correction factors for each element that account for differences between the unknown and standard in (1) the electron backscattering and energy loss (the “$Z$” factor); (2) X-ray self-absorption (the “$A$” factor); and (3) the secondary characteristic X-ray production induced by self-absorption of the primary characteristic X-rays (the “$F$’ factor).

2 $$\frac{{C_{i,sample} }}{{C_{i,standard} }} = k_{i} \cdot Z \cdot A \cdot F.$$

DTSA-II implements various different matrix correction algorithms but the default and the one used for this work is the XPP algorithm of Pouchou and Pichoir [8]. The mass absorption coefficients used are Chantler’s FFAST database [9] as made available through the NIST website. DTSA-II uses Chantler for Z<=92 and Sabbatucci’s mass absorption coefficients (MACs) [10]**.**

### Database

The database of measurements that supports this work is organized according to the measurement platform (SEM/EDS) utilized. Within the folder for each measurement platform is a DTSA-II EDS detector configuration file which incorporates the specific parameters for that detector, such as the window material and thickness. For spectrum calibration with the DTSA-II calibration tool, a folder contains Cu spectra measured at E_0_ = 20 keV or E_0_ = 15 keV, providing both the Cu L- and K- families, that were recorded over the course of the measurement campaign. A folder for each material contains the spectra that were analyzed along with the standard spectra that were utilized. A Microsoft Excel file for that material lists the results obtained with DTSA-II for each element: the raw mass concentration, the normalized mass concentration, and the atomic concentration, all with the associated uncertainty budget that includes the contributions of the measurement statistics for the sample and standard, and the estimated uncertainty in the $Z$ and $A$ matrix correction factors.

### Metric used to assess the accuracy of analysis

The measure of the accuracy of each analysis, the *relative deviation from expected value* (RDEV), has been determined as:

3 $${\text{RDEV }} = \, \left[ {\left( {{\text{measured value }}{-}{\text{ expected value}}} \right)/\left. {\text{expected value}} \right)} \right]{\text{x 1}}00\%$$

The “expected value” is taken as the elemental concentration certified for a CRM, or the ideal formula value for a stoichiometric compound. For several of the metal alloys analyzed for which independent analysis was unavailable, the expected value for the 5 keV analysis was taken as the concentration value determined at E_0_ = 20 keV using the higher-energy characteristic X-rays typically selected for conventional analysis, e.g., K instead of L, L instead of M. These higher-energy characteristic X-ray peaks are subject to a reduced uncertainty in the absorption correction and thus a reduced uncertainty budget.

## Results

Table 1 summarizes the analytical results for 263 concentration measurements for 39 elements in 113 materials measured at E_0_ = 5 keV. Listed for each element in each material are the ideal concentration (mass and atomic), the measured raw mass concentration, the normalized mass concentration, and the atomic concentration, with the RDEV values determined for each of these concentration results.

**Figure 3:**
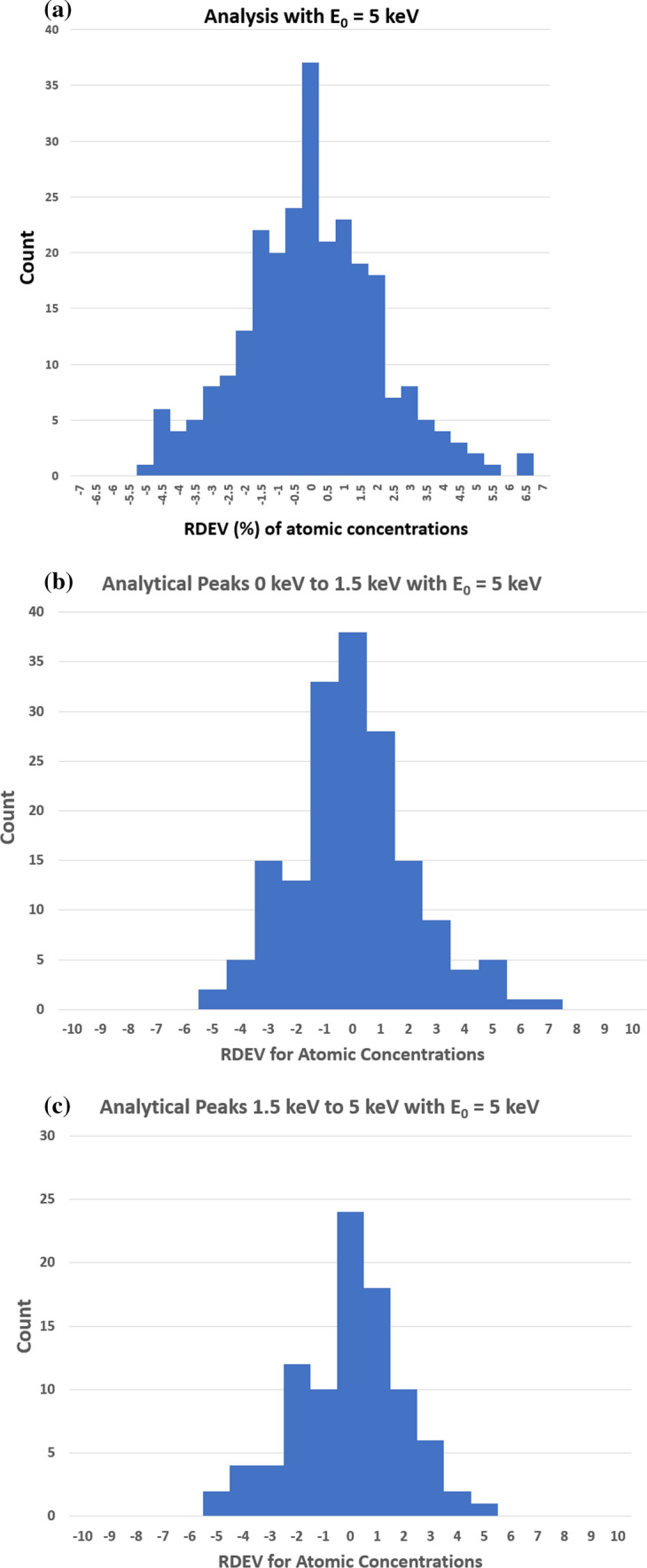
**a** Histogram of relative deviation from expected value (RDEV) for atomic concentrations from DTSA-II at E_0_ = 5 keV. **b** Histogram of relative deviation from expected value (RDEV) for atomic concentrations from DTSA-II for X-ray peak energies in the range 0–1.5 keV at E_0_ = 5 keV. **c** Histogram of relative deviation from expected value (RDEV) for atomic concentrations from DTSA-II for X-ray peak energies in the range 1.5–5 keV at E_0_ = 5 keV.

**Figure 4:**
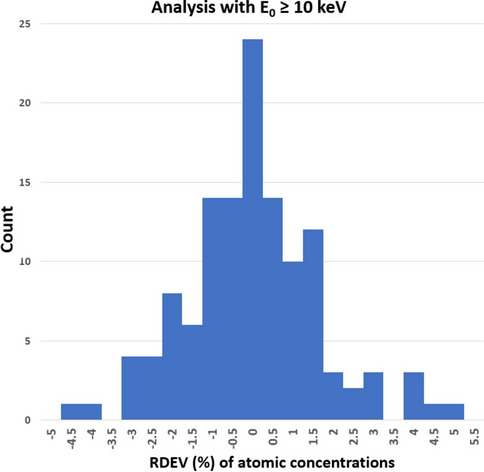
Histogram of relative deviation from expected value (RDEV) for atomic concentrations from DTSA-II for conventional analysis with E_0_ ≥ 10 keV derived from results tabulated in Newbury and Ritchie, (2015) augmented with additional analyses of II-VI and II-V compounds.

Figure 3a shows a histogram of the RDEV values at E_0_ = 5 keV for the atomic concentrations derived from Table 1. For the materials tested, the distribution of RDEV values is such that more than 98% of the results are found to be captured within a range of ±5% RDEV, and 82% of the results fall in the range − 2% to 2% RDEV. Figure 3b shows the distribution for a subset of this data for analysis of elements with characteristic X-ray peaks in the range 0–1.5 keV, and Figure 3c shows the subset for characteristic peaks in the range 1.5–5 keV. For comparison, Figure 4 shows a histogram of the RDEV values for atomic concentrations at E_0_ = 20 keV for DTSA-II analyses performed under conventional conditions with selection of the more energetic characteristic X-ray for an element whenever possible, e.g., the K-shell instead of the L-shell and the L-shell instead of the M-shell, as reported in Newbury and Richie [11]. This database was extended through the analysis of additional challenge materials recorded as part of that study, including II-VI and II-V compounds, with the spectra and DTSA-II results complied in the database associated with this publication. For this particular set of 122 elemental analyses, all of the quantitative results are captured within a range of ±5% RDEV, and 89% of the results fall in the range − 2%–2% RDEV.

**Figure 5:**
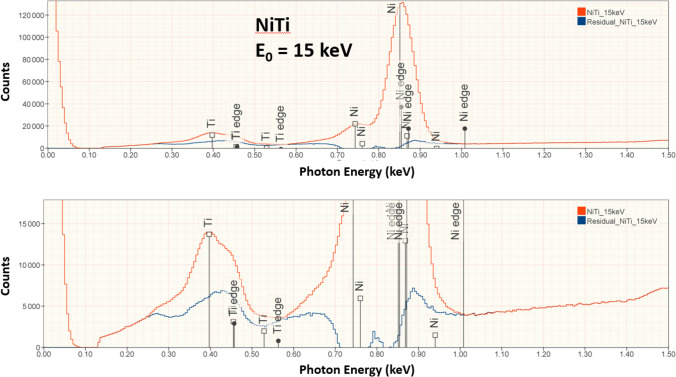
NiTi measured at E_0_ = 15 keV (red) and peak fitting residual spectrum (blue).

**Figure 6:**
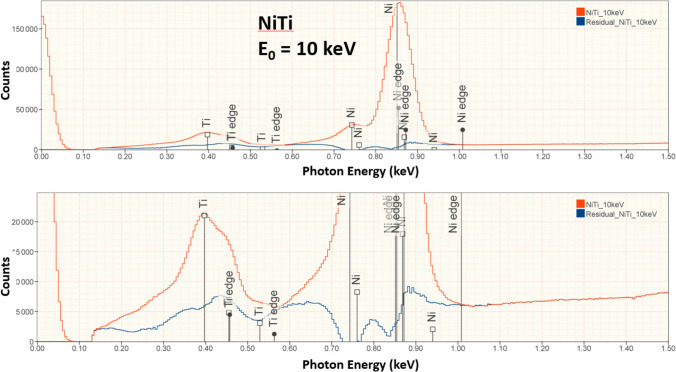
NiTi measured at E_0_ = 10 keV (red) and peak fitting residual spectrum (blue).

**Figure 7:**
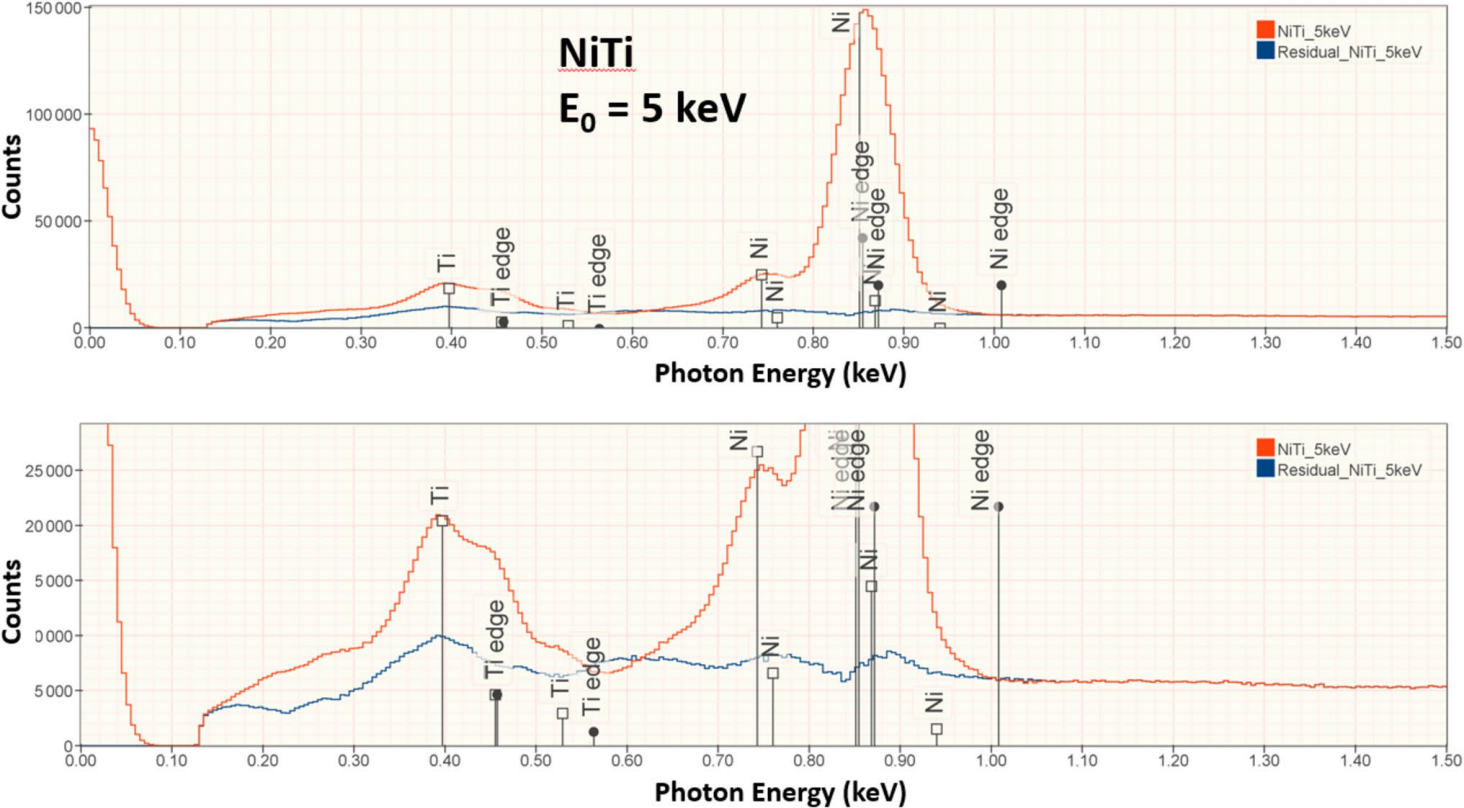
NiTi measured at E_0_ = 5 keV (red) and peak fitting residual spectrum (blue).

Figures 5, 6, and 7 show the results of fitting the characteristic X-ray peaks to reveal the X-ray continuum background that remains in the peak fitting residual spectrum in the region for the Ti L-family and the Ni L-family in EDS spectra recorded for the intermetallic compound NiTi at E_0_ = 15 keV, 10 keV, and 5 keV.

**Figure 8:**
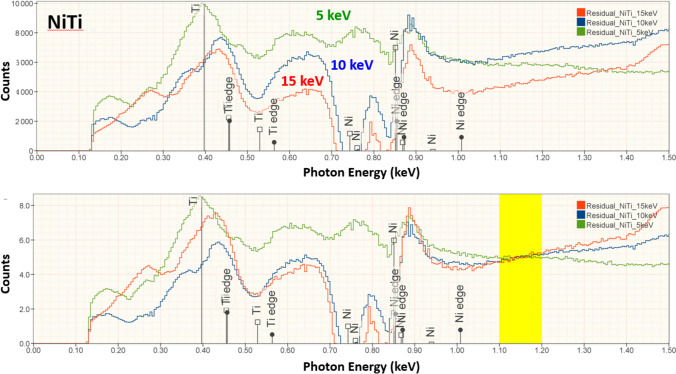
NiTi: **a** comparison of peak fitting residual spectra measured at E_0_ = 15 keV (red), 10 keV (blue) and 5 (keV (green); **b** all peak fitting residual spectra scaled to the region 1.1 keV – 1.2 keV (yellow).

Figure 8 shows a comparison of the peak fitting residual spectra of NiTi at E_0_ = 15 keV, 10 keV, and 5 keV.

**Figure 9:**
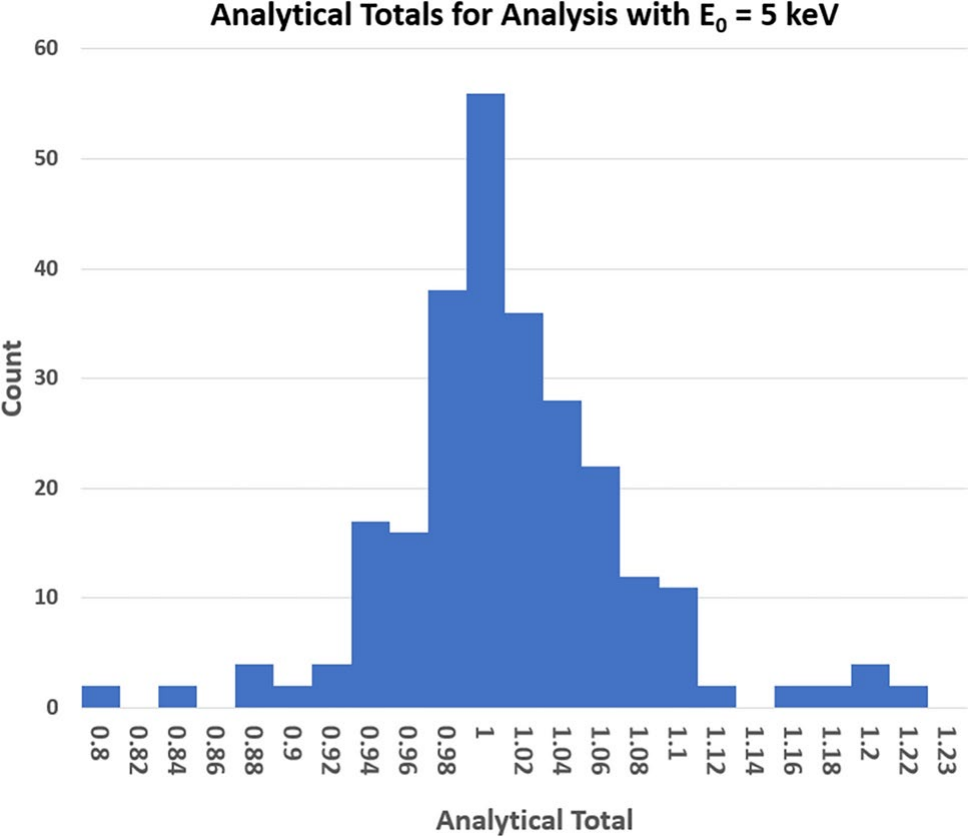
Histogram of the raw analytical total (mass concentration) from DTSA-II for E_0_ = 5 keV.

Figure 9 shows a histogram of the raw analytical totals at E_0_ = 5 keV, i.e., the sum of all constituent mass concentrations, including such as oxygen any calculated by the method of assumed stoichiometry of the cations. The distribution ranges from 0.8 to 1.2, with 67% of the analyses falling in the range 0.95–1.05.

## Discussion

The distribution of RDEV values for low-beam-energy microanalysis with E_0_ = 5 keV shown in Figure 3a is found to be similar to the RDEV distribution observed following the “conventional” analysis strategy for E_0_ ≥ 10 keV, which is shown in Figure 4 as derived from the results tabulated in Newbury and Ritchie [11], augmented with additional analyses of II-VI and II-V compounds. Conventional analysis strategy utilizes whenever possible the higher-energy characteristic X-rays that become available for certain elements with high-beam-energy excitation, e.g., the K-shell instead of the L-shell, or the L-shell instead of the M-shell. These higher-energy X-rays are subject to lower absorption, which minimizes the absorption correction and reduces the uncertainty of this often significant correction [11].

In conventional analytical practice with the beam energy E_0_ ≥ 10 keV, analysis of low-energy characteristic X-ray peaks below 1.5 keV results in larger RDEV, with the RDEV increasing as the beam energy is increased [1, 11]. However, when the RDEV distribution for analysis at E_0_ = 5 keV is examined in more detail by separately plotting the analyses for elements with characteristic X-ray peaks in the range 0–1.5 keV in Figure 3b and in the range 1.5–5 keV in Figure 3c, the RDEV distributions are found to be very similar, despite the inclusion of the low photon energy peaks (E < 1 keV) of borides, carbides, nitrides, oxides, and fluorides among the challenge compositions. Although low-beam-energy microanalysis inevitably constrains the selection of characteristic X-rays for analysis to the low photon energy X-ray family for many elements, the absorption correction for these low-energy photons is minimized at low beam energy. The reduced electron range at low beam energy significantly shortens the X-ray absorption path length, and because of the exponential dependence of X-ray absorption on the path length, the absorption correction is reduced, leading to reduced RDEV. A second advantage of reducing the self-absorption of X-rays by operating at low beam energy is the reduction in the relative height of the X-ray absorption edge structure that exists under the characteristic X-ray peaks. As the beam energy is reduced, the beam penetrates the sample less and the generated Bremsstrahlung X-rays pass through less material and fewer are absorbed on either side of the absorption edge. The magnitude of the edge structure is a function of the magnitude of the absorption which will be lower due to shorter absorption path length at lower beam energies. The result is a less distinctive absorption edge structure which is easier for the peak fitting process to handle. This absorption structure is more complex for the multi-edge L-family and M-family X-rays that must be used in low-beam-energy operation. The effect of lowering the beam energy on the quality of the peak fitting is shown in Figures 5, 6, 7, and 8 for the intermetallic compound NiTi. The “peak fitting residual spectrum” at E_0_ = 5 keV shows much reduced structure around the absorption edge energies for the Ti L-family and the Ni L-family compared to the residuals at E_0_ = 15 keV and E_0_ = 10 keV. Moreover, the residual spectrum at E_0_ = 5 keV shows a physically realistic X-ray continuum background after stripping the characteristic X-ray peaks, while the residual spectra at E_0_ = 15 keV and E_0_ = 10 keV include ranges of non-physical zero intensity in the X-ray continuum. While the residual is a very useful tool for evaluating the spectrum fit process, it should always be remembered that the residual is a somewhat artificial construct. The residual represents an estimate of the continuum signal in the unknown which is computed by subtracting, from the unknown spectrum, the characteristic intensity extracted from the standard spectrum multiplied by the k-ratio. The quality of the residual depends on many factors including the quality of the modeling of the continuum (and thus the characteristic-only intensity) in the standard, differential absorption of the various lines in the peak in the standard and sample and bonding-induced shifts in characteristic X-ray energies.

If the same low-energy lines we selected at 5 keV had been used at higher beam energies, we would expect to see less accurate results. These lines can be used at low beam energies, where the beam does not penetrate deep into the sample leading to a relatively small absorption correction despite the large mass absorption coefficients typically associated with low-energy X-rays. At higher beam energies where the beam penetrates deeper, the absorption correction and the associated uncertainty are larger resulting in significantly poorer measurements. This is particularly true for the L-lines of transition metals in which the L2-M2 transition is well known to show anomalously large self-absorption in pure metals [12–14].

The raw analytical total, which is the sum of the measured mass concentrations for all constituents, including any such as oxygen calculated by the method of assumed stoichiometry, is a useful internal consistency indicator in the conventional beam energy analysis regime, where the raw analytical total typically lies within the range 0.98–1.02 [15]. A deviation in the raw analytical total significantly below 0.98 is likely to be an indication that an element is present in the material that is not included in the element suite for the analysis, which can be a valuable tool for the analyst. The histogram of raw analytical totals observed at E_0_ = 5 keV in this study, as shown in Figure 9, reveals a much broader range. Although the majority of analytical totals cluster around unity, totals as low as 0.8 and as high as 1.2 are encountered, despite the careful vetting of the materials for surface anomalies. The source of this much broader range of analytical totals is likely to be differences in the conductive coatings applied to insulating materials. It should be noted, however, that normalization of the raw mass concentrations or calculation of atomic concentrations from the raw mass concentrations compensates for the significant deviation of the analytical total from unity. An additional factor that can affect low-beam-energy analysis is the effect of electron channeling on the characteristic X-ray emission, as reported by Meisenkothen et al. [16]. Differences between the sample and the standards in the removal of surface damage that arises from mechanical polishing, which modifies the degree of electron channeling, can influence the analytical total.

Finally, it must be remembered that the materials whose compositions were measured as challenge specimens to produce the RDEV histogram of Figure 3 were carefully examined to exclude those materials with unacceptably thick surface layers which would significantly compromise the measured composition. Thus, when considering the accuracy of low-beam-energy microanalysis applied to the analysis of practical materials, it should be noted that the reality of the sample condition that the analyst is likely to encounter on an unknown may significantly compromise the accuracy that is achievable. In particular, the analyst may encounter a surface layer, due to environmental reactions such as oxidation or sulfidation, whose thickness is a substantial fraction of the electron range. This situation creates a composite specimen where the interaction volume of the beam effectively samples two or more different materials. The basic assumption of the k-ratio protocol for quantitative analysis is that the excited volume is uniform in composition. When this condition is not met, substantial error is likely to occur. Robust analytical practice should include careful examination for the possibility of such surface anomalies by measuring spectra by sequentially lowering the beam energy from a starting value in the “conventional” range, E_0_ ≥ 10 keV, down into the low-beam-energy regime, E_0_ ≤ 5 keV, which can reveal surface anomalies, as illustrated in Figure 1.

## Data Availability

All measurements used in the preparation of this manuscript are available at Ancillary material and EDS spectra database: https://doi.org/10.18434/mds2-2853
